# Patellofemoral kinematics in dogs with cranial cruciate ligament insufficiency: an in-vivo fluoroscopic analysis during walking

**DOI:** 10.1186/s12917-017-1186-1

**Published:** 2017-08-17

**Authors:** Stanley E. Kim, Geoffrey J. Zann, Selena Tinga, Erica J. Moore, Antonio Pozzi, Scott A. Banks

**Affiliations:** 1 Department of Mechanical & Aerospace Engineering and Comparative Orthopaedics and Biomechanics Laboratory, College of Veterinary Medicine, University of Florida, PO Box 100126, 2015 SW 16th Ave, Gainesville, FL 32610-0126 USA; 2 Department of Mechanical & Aerospace Engineering, University of Florida, 231 MAE-A, PO Box 116250, Gainesville, FL 32611 USA

**Keywords:** Patellofemoral, Stifle, Kinematics, Cranial cruciate ligament

## Abstract

**Background:**

Complete rupture of the cranial cruciate ligament (CrCL) in dogs causes profound disturbance to stifle joint biomechanics. The objective of this study was to characterize the effects of cranial cruciate ligament (CrCL) insufficiency on patellofemoral (PF) kinematics in dogs during walking. Ten client-owned dogs (20-40 kg) with natural unilateral complete CrCL rupture were included. Dogs underwent computed tomographic scans to create digital bone-models of the patella and femur. Lateral projection fluoroscopy of the stifles was performed during treadmill walking. Sagittal plane PF kinematics were calculated throughout the gait cycle by overlaying digital bone models on fluoroscopic images using a previously described 2D-3D registration technique. For acquisition of kinematics in the contralateral (control) stifle, fluoroscopy was repeated 6-months after stabilizing surgery of the affected side. Results were compared between the pre-operative CrCL-deficient and 6-month post-operative control stifles.

**Results:**

Craniocaudal PF translation was similar between CrCL-deficient and control stifles throughout the gait cycle. The patella was more distal and positioned in greater flexion throughout the gait cycle in CrCL-deficient stifles when compared to the control stifle at equivalent time points. There was no significant difference in PF poses between CrCL-deficient and control stifles at equivalent femorotibial flexion angles; however, common femorotibial flexion angles were only found over a small range during the swing phase of gait.

**Conclusions:**

CrCL insufficiency altered PF kinematics during walking, where the changes were predominately attributable to the femorotibial joint being held in more flexion. Abnormal PF kinematics may play a role in the development of osteoarthritis that is commonly observed in the PF joint CrCL-deficient stifles.

## Background

Complete rupture of the cranial cruciate ligament (CrCL) in dogs causes profound disturbance to stifle biomechanics. The effects of CrCL insufficiency on stifle motion have been well characterized for the femorotibial joint, where both bench-top and in-vivo studies have shown that marked cranial tibial subluxation occurs during weight-bearing [1–5]. Patellofemoral (PF) joint abnormalities are also commonly observed in dogs with CrCL rupture, though the importance of PF pathology remains unknown. In CrCL-deficient stifles, osteoarthritis of the PF joint is typically identified radiographically [6, 7] and cartilage lesions of the PF joint have been observed on arthroscopic examination in 85–100% of dogs [8, 9].

While femorotibial instability is usually considered the predominating cause of lameness and discomfort, there is increasing interest towards understanding the pathomechanics and clinical relevance of PF joint abnormalities associated with CrCL rupture. Patellofemoral pain is common in humans, and may be a significant contributor to suboptimal limb function following anterior cruciate ligament reconstruction; in one study, the severity of PF osteoarthritis was found to be a better predictor than femorotibial osteoarthritis for outcome after anterior cruciate ligament reconstruction [10]. Discomfort arising from the PF joint is not readily recognizable in dogs, although patellar mechanism problems such as patellar tendinitis and patellar fracture are reported as painful complications of surgical stabilization of CrCL deficient stifles [11, 12].

Patellofemoral osteoarthritis seen with CrCL disease is most frequently ascribed to abnormal joint kinematics [9, 13–15]. Cadaveric investigations demonstrated altered patellar flexion angle and patellofemoral contact mechanics following CrCL transection [16, 17]. In a recent clinical study of dogs with CrCL disease, arthroscopic examination revealed that cartilage pathology was most evident in the proximal aspect of the trochlear groove [9]; these authors thus proposed that the location of cartilage lesions was consistent with the PF joint malalignment and abnormal contact mechanics described in the previously reported cadaveric study [16]. Unfortunately, the bench-top studies did not fully attempt to replicate the complex stifle kinematics occurring in-vivo; thus the potential correlation between cartilage lesion patterns seen in clinical subjects and abnormal kinematics observed in the cadaveric investigations remains unclear.

Dynamic in-vivo joint kinematics can be accurately quantified in a non-invasive manner with fluoroscopic techniques [18–22]. Using single plane fluoroscopy, our group has previously determined sagittal-plane PF kinematics in normal dogs during various daily activities [23]. We corroborated that PF kinematics are tightly coupled to femorotibial kinematics, but vary according to the type of activity performed [23]. To the authors’ knowledge, there are no in-vivo quantitative studies on PF kinematics in dogs with CrCL rupture. The purpose of this study was to characterize the disruption to PF kinematics in dogs with naturally occurring CrCL rupture during ambulation with single plane fluoroscopy. We hypothesized that in stifles affected by CrCL rupture, PF kinematics would be altered over the duration of the gait cycle in all 3 sagittal plane degrees of freedom when compared to normal stifles; specifically, we predicted there would be less ‘wrapping’ of the patellar /patellar tendon mechanism on the femoral trochlea, thereby causing the patella to ride more cranially and proximally, and be carried in less flexion.

## Methods

Adult, non-chondrodystrophic dogs weighing 20 to 40 kg with naturally occurring unilateral complete CrCL rupture were recruited for the study. Orthopedic examination was performed by a board certified surgeon. Inclusion criteria consisted of: unilateral complete CrCL rupture as determined by palpable craniocaudal stifle instability (unilateral positive cranial drawer sign and positive tibial compression test); no concurrent stifle abnormalities such as medial patellar luxation; tibial plateau angle ≤35°; and duration of lameness <6-months duration. Upon assessment with the orthopaedic exam and fluoroscopic analysis, the dogs had to be consistently weight-bearing on the affected limb during walking. Diagnosis was confirmed at the time of surgery. The contralateral stifle had to be palpably stable, without pain or palpable effusion, and free of radiographic signs of osteoarthritis over the duration of the study. All affected stifles were treated by tibial plateau leveling osteotomy (TPLO), as previously described [24]; PF kinematics following TPLO treatment will be described in a separate report. The study was approved by the University’s Institutional Animal Care and Use Committee and owners signed informed consent at the time of enrolment.

### Fluoroscopic image acquisition

Prior to surgery, horizontal-beam lateral projection fluoroscopic images of the stifles were acquired^1^ while the dog walked on a treadmill at a velocity of 2.0–2.5 mph (0.8–1.1 m/s) that allowed a natural walking cadence. Approximately 15 full gait cycles were collected over 3 separate trials. Images were acquired using a pulse width of 1 ms at 30 frames/s. Three representative gait cycles were chosen for processing.

### 3-dimensional model creation

**Fig. 1:**
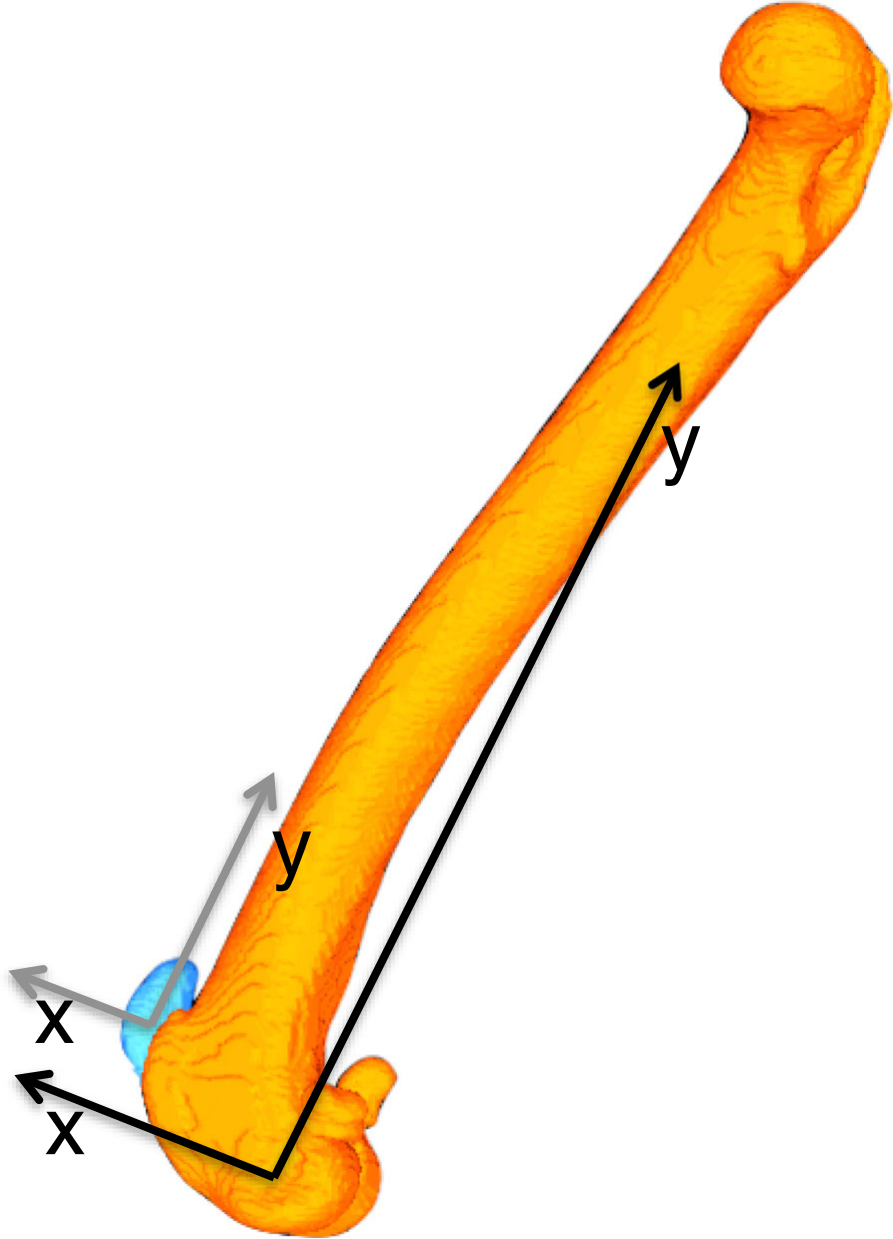
Femoral and patellar coordinate systems. Patellar coordinates are indicated in grey; femoral coordinates indicated in black

Prior to surgery, computed tomographic scans^2^ with a 512 × 512 image matrix, a 0.35 × 0.35 pixel dim, and 0.5 mm slice thickness with 0.3 mm overlap were obtained over the full length of the femora, including the patellas. Three-dimensional digital bone models of the affected and contralateral femurs and patellas were created; the cortical bone margins were segmented using an open source 3D segmentation software program^3^, and these point-clouds were converted into polygonal surface models with a reverse engineering software program^4^. Anatomic coordinate systems were applied to the bone models based on anatomical landmarks of the patella and femur [23]. Femoral coordinates were applied such that the mediolateral axis (z-axis) passed through the center of the lateral and medial femoral condyles with the femoral origin located at the mid-point between the condyles (Fig. 1). The proximodistal axis (y-axis) passed proximally along the femoral shaft, perpendicular to the mediolateral axis in the plane common to the center of both femoral condyles and the femoral head. Patellar coordinates were applied such that the mediolateral axis (z-axis) passed through the most medial and lateral points of the bone with the patellar origin located at the mid-point between these points. The proximodistal axis (y-axis) passed proximally, perpendicular to the mediolateral axis, passing through the most proximal aspect of the patella. The craniocaudal axes (x-axis) for the femur and patella were created from the cross product of the mediolateral and proximodistal axes, thus creating Cartesian coordinate systems in each bone.

### 3-dimensional to 2-dimensional image registration

**Fig. 2:**
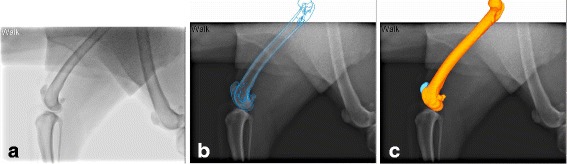
Image registration process. **a** Flat-panel radiographic image imported into JointTrack^e^. **b** Contoured silhouettes based on the 3-D models of the femur and patella utilized for shape-matching over the radiographic image. **c** Shape-matched 3-D projections of the femur and patella

To ascertain PF kinematics, a 3D–to-2D image registration process was used to combine 3-dimensional bone model data with 2-dimensional fluoroscopic data from 3 representative gait cycles that best caught the entire gait cycle encompassed within the fluoroscopic field of view. The digital bone models of the patella and femur were superimposed over the fluoroscopic images and their projected silhouettes were translated and rotated such that the contours precisely matched the corresponding contours of the fluoroscopic images^5^, as previously described (Fig. 2) [23]. The patella was positioned centrally within the trochlear groove, such that the articulating surface of the patella at the center of the patella remained as congruent as possible with the trochlear groove in the axial plane. The output of the software represents the individual model positions in space, and these results were converted to the relative positions of the bone models to each other using a custom computer program^6^.

### Control kinematic data

Contralateral limb kinematics have been shown to be affected by the presence of lameness caused by CrCL insufficiency [25]; therefore, data for the contralateral limb was collected and evaluated 6-months following TPLO of the CrCL-deficient limb to represent the control values. A prior study has found no difference in force plate analysis between 6-month post-operative TPLO-treated naturally affected CrCL-deficient dogs compared to control dogs, indicating that this time frame should allow return to soundness [26]. No lameness was evident in either hind limb for all dogs at this time point.

### Kinematic data processing

Data were split into stance and swing phases and each phase was time normalized using a custom spline interpolation program^7^ at 1% intervals to allow averaging between dogs despite temporal differences. Kinematic data was compiled for patellar craniocaudal translation, proximodistal translation, and flexion/extension angle for both the pre-operative CrCL-deficient stifle and the 6-month post-operative contralateral stifle (internal control). The PF kinematic parameters were described both temporally and in relation to femorotibial flexion/extension angles determined from data acquired in a companion study [5].

### Statistical analysis

A statistical package was used for all analyses^8^. The data from 3 gait cycles was averaged to calculate mean values at each time point in the gait cycle for each dog. Paired T-tests were used to compare PF craniocaudal translation, proximodistal translation, and flexion/extension between the CrCL-deficient and control stifles at 10% increments over both stance and swing phase of gait. Comparisons between CrCL-deficient stifles and control stifles encompassed the entire gait cycle, including early and late stance phase of gait, as well as early and late swing phase of gait. PF poses are strongly influenced by femorotibial flexion angle [17]; thus, to elucidate for changes to PF kinematics occurring independent of differences in femorotibial flexion angle, each PF kinematic parameter was compared between CrCL-deficient and control stifles at common femorotibial flexion angles over 5**°** increments. For all statistical analyses performed, *P* < 0.05 was considered statistically significant.

## Results

Ten dogs were included in the study. Five were mixed breed dogs, 2 were Labrador Retrievers, and the remaining dogs consisted of 1 Standard Poodle, 1 English Springer Spaniel, and 1 Siberian Husky. Five were spayed females and 5 were castrated males. Mean ± SD age was 6.6 ± 2.8 years. Mean ± SD body weight was 30.3 ± 6.0 kg with a median body condition score of 6/9 (range 4–8). The right stifle was affected in 7 dogs and the left in 3 dogs. Mean ± SD duration of lameness prior to presentation was 2.3 ± 2.1 months. Mean ± SD tibial plateau angle was 29.4 ± 3.1^o^ for the CrCL-deficient stifle and 28.5 ± 3.4^o^ for the contralateral control stifle (*P* = 0.26). Meniscal pathology was not identified in 4 dogs, while 6 dogs had injury to the caudal pole of the medial meniscus that required debridement. No dogs suffered any complications or had unsatisfactory outcomes following TPLO; all dogs were fully weight-bearing on all four limbs during ambulation at 6-months after TPLO, based on visual assessment by a board certified surgeon (SEK).

**Fig. 3:**
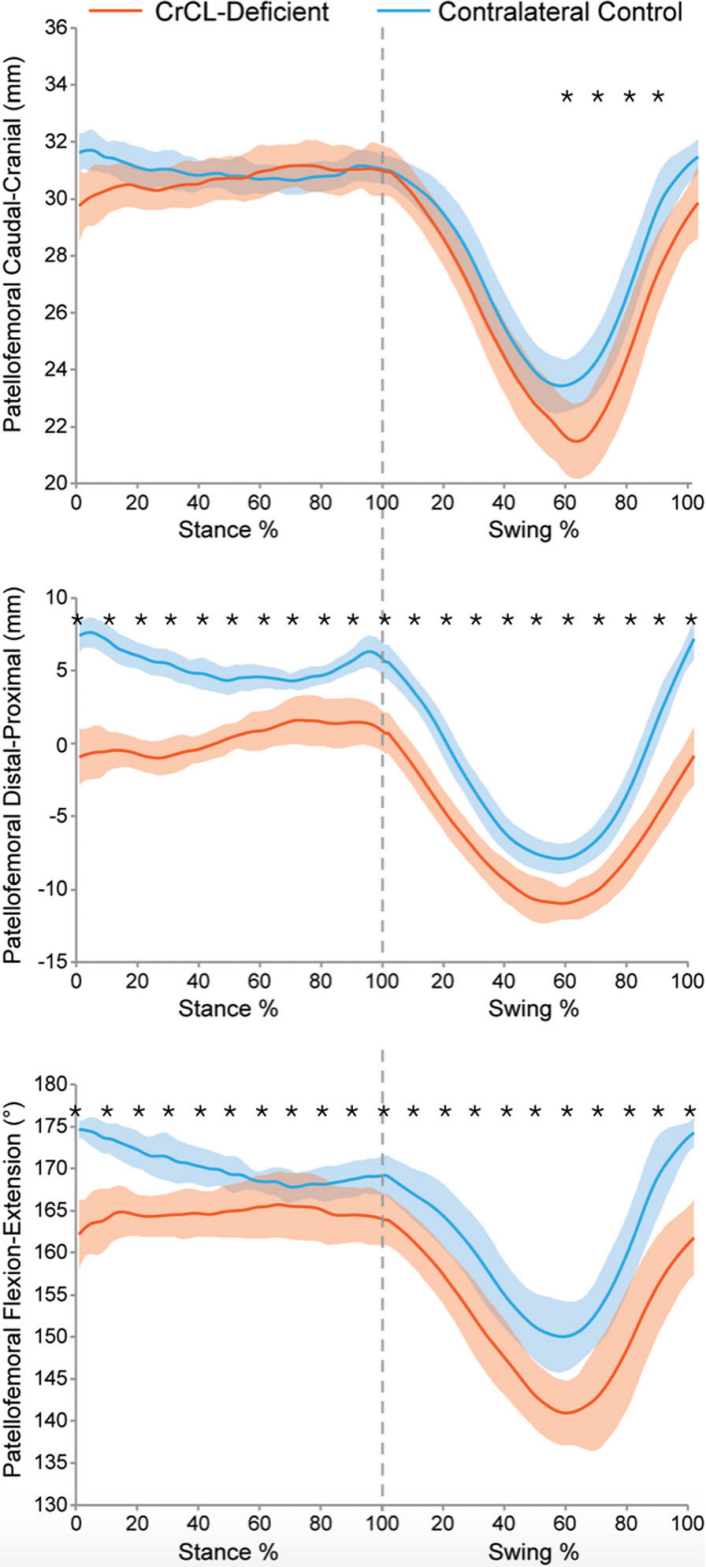
PF craniocaudal translation (**a**), proximodistal translation (**b**), and flexion-extension (**c**), over the entire gait cycle. Orange line = CrCL-deficient, Blue line = control. Solid line = swing phase, dotted line = stance phase. Lines represent the mean values for all dogs; The shaded areas represent standard deviation and * represents a statistically significant difference at that time point. There was no significant difference in craniocaudal translation throughout stance phase between CrCL-deficient and control stifles, but the patella was more caudal in CrCL-deficient stifles when compared to control stifles in part of swing phase. The patella was more distal and held in greater flexion in CrCL-deficient stifles when compared to control stifles throughout the entire gait cycle

In both CrCL-deficient and control stifles, PF joint excursions were nominal during the stance phase and largest during the swing phase of gait for all 3 kinematic parameters (Fig. 3). During the first half of swing phase, the patella translated caudodistally and rotated into greater flexion; during the second half of swing phase, the patella translated proximocranially and rotated into greater extension.

There was no significant difference in craniocaudal translation throughout stance phase between CrCL-deficient and normal stifles, but the patella was more caudal in CrCL-deficient stifles when compared to normal stifles in most of swing phase (Fig. 3). The patella was more distal in CrCL-deficient stifles when compared to normal stifles throughout the entire gait cycle. The patella was also held in greater flexion throughout the entire gait cycle in CrCL-deficient stifles when compared to normal stifles.

**Fig. 4:**
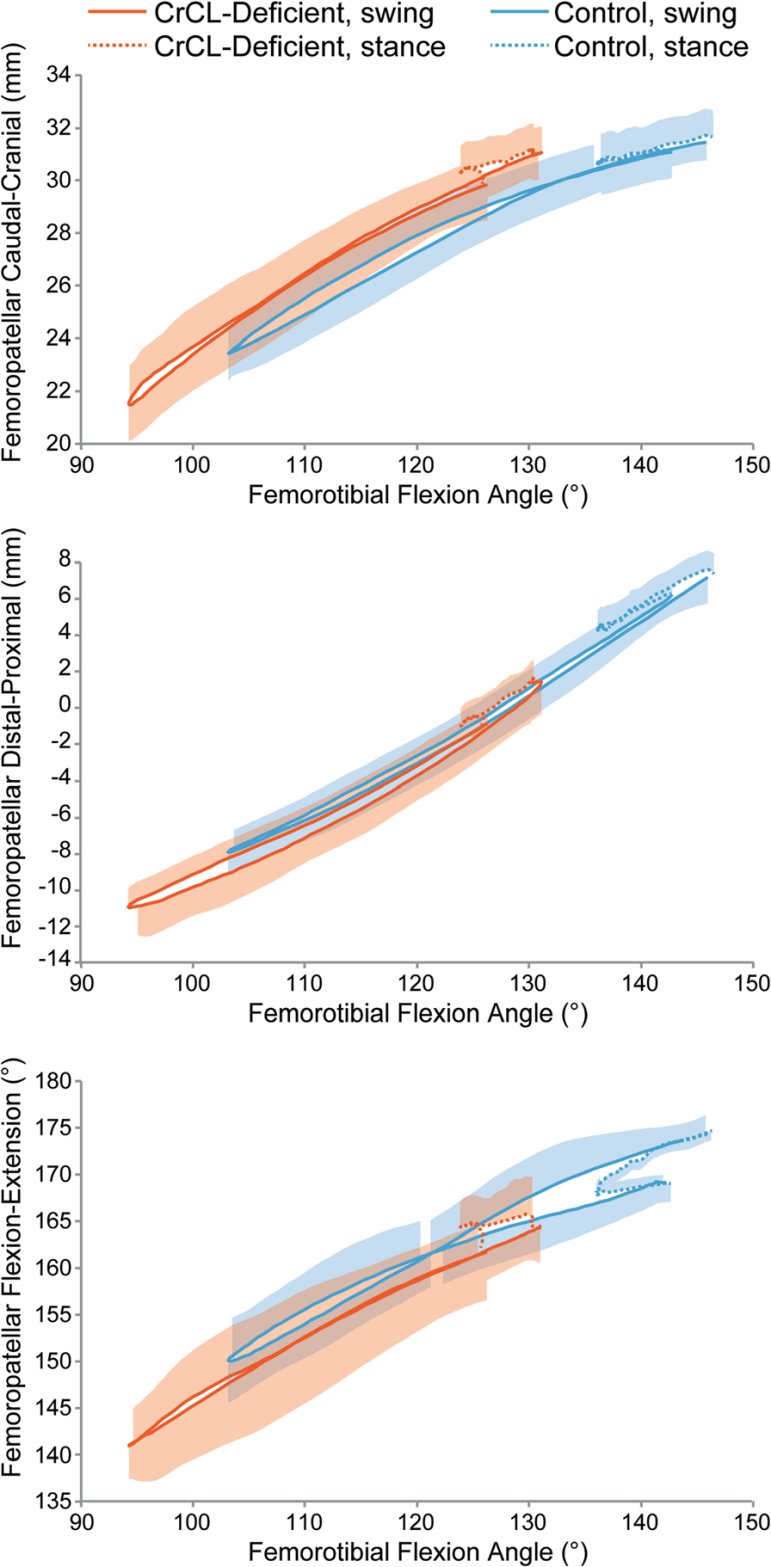
Averaged plots of femorotibial flexion angle vs PF flexion angle during walking. For both femorotibial and PF flexion angle, greater values indicate greater extension. There was no difference in PF flexion at equivalent femorotibial flexion angles

When plotted against femorotibial flexion angle, there was no significant difference between CrCL-deficient and control stifles for PF craniocaudal translation, flexion extension, and proximal-distal translation at equivalent femorotibial flexion angles (Fig. 4); however, common femorotibial flexion angles were only found over a 26° range (out of a possible 50° degree range) during swing phase of gait, and there were no common femorotibial flexion angles for stance phase of gait.

## Discussion

This in-vivo study characterized sagittal-plane kinematics of the PF joint in stifles affected with naturally occurring CrCL rupture. Abnormal poses were evident in CrCL-deficient stifles during walking: the patella was held in greater flexion and more distally when compared to control stifles throughout both stance and swing phases of gait; however, craniocaudal translation of the patella in CrCL-deficient stifles was mostly similar to control stifles. Our findings corroborate the intuitive relationship between PF kinematics and femorotibial flexion angle, but we also identified disruption to normal PF kinematics that could not be directly attributed to femorotibial flexion angle.

When assessed according to progression through the gait cycle, CrCL rupture did not appear to substantially influence craniocaudal patellar translation during the stance phase. This should not be interpreted in isolation, and our data actually demonstrates craniocaudal patellar translation may have been affected by CrCL rupture with 2 distinct mechanisms that essentially ‘cancelled’ each other out. Greater flexion of the femorotibial joint generally caused greater caudal patellar translation in both CrCL-deficient and control stifles. Because CrCL-deficient stifles are held in greater femorotibial flexion over the gait cycle [5], the patella is expected to be more caudally positioned in CrCL-deficient joints at equivalent time points over the gait cycle; however, no significant difference in craniocaudal PF translation was found between CrCL-deficient and control joints during stance phase. We suspect this lack of difference was the result of cranial tibial subluxation across the femorotibial joint that is most prominent during stance phase [5], which would shift the patellar tendon and patella cranially. Weakened quadriceps muscles may also play a role, where the patella fails to adequately compress into the trochlear groove due to poor quadriceps tone [27].

We found that the patella was distally displaced in CrCL-deficient joints when compared to normal joints throughout the entire gait cycle. Distal patellar translation was most likely induced by the greater flexion of the femorotibial joint occurring throughout ambulation. In one cadaveric experiment, CrCL transection resulted in a proximal shift of PF joint contact area [16]; however, femorotibial flexion angle in that study was kept constant between normal and CrCL-deficient conditions. Our findings conflict with a proposed theory that the cartilage lesions that are commonly identified in the proximal trochlea with CrCL rupture are induced by excessive wear from the proximal shift of the patellofemoral contact regions [9]. As cartilage requires physiologic mechanical loading to maintain optimal health, under-stressed regions of joints are also susceptible to cartilage lesions [15]; given that we found the patella was distally displaced throughout the gait cycle, the proximal cartilage lesions may potentially be the result of inadequate PF contact in those regions. While it is possible that certain daily activities cause the patella to track more proximally with CrCL insufficiency when compared to normal, a previous study of normal dogs found no difference in greatest femorotibial extension between walking, trotting, and stair-climbing [28]. The relationship between PF kinematics and cartilage wear is currently unclear, as precise anatomic localization along the trochlear groove cannot be correlated between studies.

The patella was maintained in greater flexion in CrCL-deficient joints throughout both stance phase and swing phase of gait. Patellar flexion angle appeared to be predominately dependent on femorotibial flexion angle; because the femorotibial joint is carried in more flexion during walking with CrCL rupture [5], observing greater patellar flexion in affected stifles was not surprising. A radiographic investigation performed in canine cadaveric specimens found patellar flexion increased slightly with CrCL-deficiency when the joint was consistently forced into subluxation, over a wide range of femorotibial flexion-extension motion [17]. Our results conflict with those findings, where we found that the relationship between patellar flexion and femorotibial flexion appeared tightly constrained in an equivalent pattern for both CrCL-deficient and control joints.

While we were able to accurately quantify abnormal PF kinematics induced by CrCL insufficiency, the clinical relevance of the changes remains unknown. Because osteoarthritis is often considered to be a mechanically induced disorder, it is tempting to ascribe PF osteoarthritis to the kinematic disturbances we characterized; however, a direct causal relationship between abnormal kinematics and osteoarthritis cannot be made from this study. Future in-vivo investigations describing joint loading patterns, accurate mapping of cartilage lesions, and attempts at quantifying clinically relevant morbidity from PF osteoarthritis would be required to gain further insight into PF abnormalities associated with CrCL insufficiency.

There were numerous limitations to our study. The single-plane fluoroscopic methodology restricted the analysis to sagittal plane kinematics only. Several studies of humans with anterior cruciate ligament rupture have demonstrated more pronounced PF kinematic changes in the coronal plane (patellar tilt, medial-lateral translation) [29, 30], which could not be assessed in our study. Additionally, we only evaluated dogs during walking, and it is possible that the most relevant disturbances to PF kinematics are present during other daily activities. It is possible that the PF joint was not normal prior to the development of CCL insufficiency; however, we were not able to assess the pre-injury state with our study. We also used PF kinematics of the contralateral stifle as the control, which may have been influenced by gait disturbances of the diseased hind limb. To mitigate this potential issue, control PF kinematics were quantified when the CrCL-deficient limb was clinically sound 6-months after treatment by TPLO.

## Conclusions

This study demonstrated that CrCL insufficiency alters PF kinematics during walking, and the most profound changes were attributable to the femorotibial joint being held in more flexion throughout the gait cycle, and cranial tibial subluxation during stance phase of gait. The abnormal PF kinematics we identified may play a role in the development of PF osteoarthritis commonly observed in dogs with CrCL insufficiency. The ideal treatment of CrCL insufficiency should therefore aim to restore both tibiofemoral and PF kinematics to normal.

## Abbreviations

CrCL: Cranial cruciate ligament
PF: Patellofemoral
TPLO: Tibial plateau leveling osteotomy

## Footnotes

1 Toshiba American Medical Systems, Inc., Tustin, CA.

2 Toshiba Aquilon 8, Toshiba American Medical Systems, Inc., Tustin, CA.

3 ITK-SNAP, http://www.itksnap.org.

4 Geomagic, Inc., Research Triangle Park, NC.

5 JointTrack, University of Florida: http://sourceforge.net/projects/jointtrack/.

6 MATLAB and Statistics Toolbox Release R2015a, The MathWorks, Inc., Natick, MA.

7 MATLAB and Statistics Toolbox Release R2015a, The MathWorks, Inc., Natick, MA

8 SigmaPlot 12, Systat Software, Inc., San Jose, CA.
